# Pediatric Traumatic Limb Amputation: The Principles of Management and Optimal Residual Limb Lengths

**Published:** 2016-01

**Authors:** Muhammad Adil Abbas Khan, Ammar Asrar Javed, Dominic Jordan Rao, J Antony Corner, Peter Rosenfield

**Affiliations:** 1 St. Mary’s Hospital, London, UK;; 2 The Johns Hopkins Hospital, Baltimore, USA

**Keywords:** Pediatric, Trauma, Amputation, Residual limb length

## Abstract

Pediatric traumatic limb amputations are rare and their acute and long term management can be challenging in this subgroup of patients. The lengthy and costly hospital stays, and resulting physical and psychological implications leads to significant morbidity. We present a summary of treatment principles and the evidence base supporting the management options for this entity.

The initial management focuses on resuscitating and stabilization of the patients, administration of appropriate and adequate analgesics, and broad spectrum antibiotics. The patient should ideally be managed by an orthopedic or a plastic surgeon and when an amputation is warranted, the surgical team should aim to conserve as much of the viable physis as possible aimed at allowing bone development in a growing child. A subsequent wound inspection should be performed to assess for signs of ischemia or non-viability of tissue. Depending on the child’s age, approximations of the ideal residual limb length can be calculated using our guidelines, allowing an ideal stump length at skeletal maturity for a well-fitting and appropriate prosthesis. Myodesis and myoplasties can be performed according to the nature of the amputation.

Removable rigid dressings are safe and cost effective offering better protection of the stump. Complications such as necrosis and exostosis, on subsequent examination, warrant further revisions. Other complications such as neuromas can be prevented by proximal division of the nerves. Successful rehabilitation can be accomplished with a multidisciplinary approach, involving physiotherapist, play therapist and a child psychiatrist, in addition to the surgeon and primary care providers.

## INTRODUCTION

Trauma is ranked worldwide in the top causes of mortality and morbidity in children over the age of one year.^1^ Pediatric limb amputations, despite being rare are a unique and account for a disproportionately large component of the morbidity associated with trauma.^2^ These amputations are not amenable to replantation and very little literature is available to assist surgeons in their overall management. This article aims to give an overview of the important surgical and non-surgical aspects of the management of these cases and also identifies useful tools to help modifying the available management aimed at better, uneventful recovery of the patient. 

While there is a general lack of literature on their prevalence, pediatric traumatic limb amputations are more common and extensive when compared to adults.^3^ 111600 children with amputations were treated in US emergency departments alone from 1990 to 2002.^4^ Of these, the majority occurred around 1 year of age with a steady decline seen up to 7 years of age.^4^ In one review by Trautwein et al. on pediatric trauma patients, it was seen that 22% were caused by lawn mower injuries and 16% by motor vehicle related injuries.^2^ All patients that sustained lawn mower related injuries had amputations as compared to 77% of motor vehicle injuries that resulted in amputations.^2^

Traumatic limb amputations are serious injuries that can result in prolonged hospital stays, disability and psychological consequences for the amputee.^3^ This can complicate the recovery of the child leading to problems including depression, confusion, problems with everyday activities, phantom limb sensations and pain, all of which affect their future goals.^5^ In addition, the significant amount of time and perseverance required from the parents, the hospital staff and the physiotherapist can be overwhelming. From a financial perspective, these amputations are costly to treat. Trautwein et al. in their study of 74 patients, reported a mean stay of 11.3 days with an average of 4 procedures, costing up to $22,015 2. Similar results were reported in a study on lawnmower injuries by Loder et al.^6^ Also in centers where referral facilities are not available, the stay can be prolonged till transfer to a suitable specialist center can be arranged.

Developments in the assessment and management of pediatric trauma have been minor as compared to those in adult trauma given that the number of critically injured children is smaller in this population.^7^ Pediatric trauma centers have been developed based on the belief that care of pediatric trauma patients differs significantly from that provided to adults and approach for the pediatric population should be individualized and personalized. Evidence supporting improved survival rates in children brought to a pediatric trauma center as opposed to a community hospital is controversial, thus the prohibitive cost of setting up a dedicated pediatric trauma facility has seen the development of only a limited the number of such centers.^8^ Despite this the argument, that a small yet substantial number of dedicated pediatric trauma centers is a cost-effective and efficient way to provide the multidisciplinary environment needed for the management of these cases, is indeed a valid one.

*Initial Evaluation and Resuscitation*

The initial management of a pediatric trauma patients focuses on the principles of Advanced Trauma Life Support (ATLS) and Advanced Pediatric Live Support (APLS) that is to stabilize the patient, focusing on maintaining the airway, breathing and circulation.^9^ Other variables to consider are summarized in Table 1. There are various available objective clinical scoring systems which can be used in the assessment of trauma patients and can help the surgical team. The systems applicable to the pediatric population include the Mangled Extremity Severity Score (MESS), the Pediatric Trauma Score (PTS) and the Injury Severity Score (ISS) (Table 2). MESS is a valid and reproducible scoring system that uses an objective criterion to assist with the decisions of acute management for a primary amputation with an open fracture and mangled extremity.^10^ Numerical values are assigned based on factors including age, limb ischemia, shock and skeletal soft tissue injury; a score of 7 points or higher indicates the need for amputation. Lack of or compromised perfusion of the limb for more than six hours is an important factor, doubling the score. In one study where MESS was used to classify patients into limb salvage and amputation groups, its prediction for the salvage group was accurate in 93% of the injured limbs as compared to 63% in the latter group.^11^ However, each case needs to be handled on an individual level and the MESS score should only be used as a guide and not the principal determinant for performing an amputation.

**Table 1:** Important points to consider in the specific management of pediatric amputations

Important points in amputation
Initial patient’s assessment (ABC), resuscitation, transfusion protocols and hemostasis
Wound management (swabs, lavage, dressings) and stump protection
Limb splinting (in cases of partial amputation)
Antibiotics and tetanus Prophylaxis
Appropriate transport of the amputated limb
Appropriate imaging of the stump and limb (X-ray, CT)

**Table 2:** The objective scoring systems which can be used in the assessment of trauma patients

**Mangled Extremity Severity Score**	**Pediatric Trauma Score**	**Injury Severity Score**
Mechanism of injury Low energy (stabs, simple closed fracture, small-caliber gunshot) 1 points Medium energy (Open or multi-level fracture, dislocation, moderate crush) 2 points	Weight > 20 kg +2 points 10 – 20 kg +1 point < 10 kg -1 point	Abbreviated injury Scale (AIS)
High energy (Close range shotgun, high velocity gunshot) 3 points Massive crush (logging, railroad, oil rig accidents) 4 points	Airway Patent +2 points Maintainable +1 point Unmaintainable -1 point	Region Head and neck Face Chest Abdomen Extremity External
Shock Normotensive (BP stable in field and in OR) 0 points Transiently hypotensive (BP unstable in field, but responsive to IV fluids) 1 points Prolonged hypotension (Systolic BP under 90 mmHg in field and responsive only to fluids in ER) 2 points	Systolic BP > 90 mmHg +2 points 50 - 90 mmHg +1 point < 90 mmHg -1 point	AIS Score of injury: Minor 1 Moderate 2 Serious 3 Severe 4 Critical 5
Limb ischaemia None (pulsatile limb w/o ischemia) 0 points Mild (Diminished pulses w/o signs of ischemia) 1 points Mild and prolonged (same as mild above, but over 6 hours duration) 2 points Moderate (No pulse by doppler, sluggish cap refill, paresthesia, weak) 2 points Advanced (Pulseless, cool, paralyzed, numb, no cap refill) 3 points Moderate and prolonged (same as moderate above, but over 6 hours duration) 4 points Advanced and prolonged (same as advanced above, but over 6 hours duration) 6 points	CNS Awake +2 points LOC +1 point Unresponsive -1 point Fractures None +2 points Closed or suspected +1 point Multiple closed or open -1 point	Untreatable 6 Each injury refers to area with severity ranking. Only the highest AIS score is used for each body region. Three most severely injured body area scores are squared and added together (Injury Severity Score). ISS values 0 – 75.
Age under 30 years 0 points 30-49 years 1 points over 50 years 2 points	Wounds None +2 points Minor +1 point Major, penetrating or burns -1 point Mortality is estimated at 9% with a PTS > 8, and at 100% with a PTS ≤ 0. There is a linear relationship between the	
Assessment 0-6 probably viable limb >6 very high likelihood of amputation	mortality risk (i.e. the lower the PTS, the higher the mortality risk). The minimal score is -6 and the maximum score is +12.	

*Classification*

A review of literature revealed the lack of a classification system for pediatric traumatic amputations. They may be classified as partial or complete according to the extremity involved. Given that the extent of soft tissue injury is often underestimated on the initial examination, Faraj et al. proposed a peri-operative classification that covers extent of soft tissue injury and skeletal stability and predicts the outcome more accurately than the Gustilo classification.^12^ Traumatic amputations, like all open fractures, require surgical debridement within six hours of the injury to preserve soft tissue viability and prevent infections. However, since pediatric patients have greater wound healing ability, a more conservative approach to debridement may become possible with only grossly non-viable tissue removed during the first surgical intervention.^11^

If amputation becomes necessary it is of utmost importance that the resection of all devitalized soft tissue and bone is performed. The length of the stump is of vital importance for later rehabilitation and the surgeon’s awareness of the differential bone growth rates of multiple long bones can help engineer a more conservative approach to salvage as much of the bone, muscles and other soft tissues as possible without compromising adequacy of debridement. The physis plays a pivotal role in a growing limb, and hence all possible effort should be made to preserve it. This can have far reaching consequences for later rehabilitation and prosthesis fitting.^13^ A second wound inspection to assess for signs of ischemia or non-viability of tissue and further debridement should ideally be conducted at 24 to 48 hours post-operatively. While performing a primary closure has been suggested in traumatic below-knee amputations if the time from injury is less than 6 hours, the general trend is to delay and undertake secondary closure after re-examination in the operating room.^12^^,^^14^

Herscovici et al. recommended that vacuum assisted closure (VAC) be applied as a temporary adjunct for the treatment of high energy trauma.^15^ VAC therapy has been associated with a decreased frequency of dressing changes and reduced tissue edema.^16^ Measurement of skin oxygen saturation (SO2) can also be used to predict the viability of tissues in lower limb amputations by assessing limb ischemia.^17^

*Principles of Optimal Residual Limb Lengths*

For an upper limb transhumeral amputation, the objective is to achieve a 10 cm clearance between the bone-end to elbow joint axis by the end of skeletal maturity (Table 3); thus in such a case, the recommended bone section would be the junction of middle and lower third of humerus. The minimum recommended length would be at the surgical neck of the humerus where the amputation would be treated as a shoulder disarticulation. This is preferred to a shoulder disarticulation through the joint. The end of the bone is ideally chamfered circumferentially and the nerves are trimmed proximally to allow sufficient retraction away from the site of amputation. Both the biceps and triceps can be sutured to the bone end via drill holes and myofascial closure is attempted over this myodesis. The skin flaps should be equal on both the anterior and posterior aspect. (Table 4).

**Table 3:** Amputation levels for optimal residual limb lengths for the humerus, radius and ulna.^36^

	**Girls (Age in Years)**										
	**7**	**8**	**9**	**10**	**11**	**12**	**13**	**14**	**15**		
Humerus	21.9	23.3	24.4	25.6	27.0	28.7	30.2	31.2	31.6		
Radius	16.3	17.2	18.1	18.9	20.0	21.3	22.4	23.1	23.4		
Ulna	17.4	18.4	19.4	20.4	21.5	23.0	24.0	24.8	25.1		
Stature	123.1	129.5	135.8	141.2	147.5	155.6	162.4	165.2	167.4		
	**Boys (Age in Years)**										
	**7**	**8**	**9**	**10**	**11**	**12**	**13**	**14**	**15**	**16**	**17**
Humerus	22.8	23.5	24.6	25.8	27.0	28.2	29.7	31.3	32.9	34.1	34.7
Radius	16.6	17.5	18.4	19.3	20.3	21.2	22.4	23.7	24.9	25.8	26.2
Ulna	17.3	18.3	19.4	20.2	21.3	22.5	23.8	25.2	26.5	27.5	27.9
Stature	122.3	128.2	144	139.4	144	149.0	155.4	162.9	169.8	175.2	177.4

**Table 4:** Optimal residual limb lengths based on the level of amputation

**Level of** **amputation**	**Recommended bone** **section**	**Coverage**	**Comments**
Transhumeral	10 cm between bone end to elbow joint	Perform myofasical closure over myodesis	Bone ends should be chamfered
Transradial	14 cm from olecranon	Myodesis of flexor and extensor muscles with myofascial closure	
**Transfemoral**	14 cm proximal to the medial knee joint line	Myodesis and myoplasty over bone end	Bone end Circumferentially rounded
**Transtibial**	15 cm from the medial knee joint line	Myodesis of calf muscles to the periosteum	Tibial crest is chamfered

If the upper limb amputation is performed at the transradial level, the ideal residual limb length at skeletal maturity should be 14cm from the olecranon (Table 3). The shortest functional residual limb should be 2-3 cm with intact biceps and triceps tendons. Myodesis of the flexor and extensor muscles is performed with myofascial closure. The anterior and posterior skin flaps should again be equal (Table 4).

In the case of a lower limb transfemoral amputation the ideal level of bone section would be 14 cm proximal to the medial joint line (Table 5). The bone end should be circumferentially well rounded. A first layer of myodesis is performed using drill holes and a second layer of myoplasty over the bone end follows. The sciatic and femoral nerves are to be trimmed proximally to allow retraction into the soft tissue away from the amputation site. The artery associated with the sciatic nerve is to be ligated separately (Table 4).

**Table 5:** Variation in yearly rates of growth at consecutive chronological ages for the femur and tibia.^37^

**Girls (N=50)**						**Age intervals (Years)**	**Boys (N=50)**					
**Stature**		**Femur**		**Tibia**			**Stature**		**Femur**		**Tibia**	
**Mean**	**σ**	**Mean**	**σ**	**Mean**	**σ**		**Mean**	**Σ**	**Mean**	**σ**	**Mean**	**σ**
5.7	0.77	2.0	0.28	1.7	0.29	8-9	5.7	0.88	(2.0)	(0.27)	(1.6)	(0.22)
6.0	1.39	2.0	0.32	1.8	0.36	9-10	5.2	0.91	(1.8)	(0.32)	(1.5)	(0.27)
6.7	1.70	2.1	0.35	1.8	0.38	10-11	5.0	0.80	1.8	0.34	1.5	0.28
6.5	1.91	1.9	0.52	1.6	1.56	11-12	5.9	1.60	1.9	0.42	1.7	0.42
5.2	2.20*	1.4	0.67	1.0	0.63	12-13	6.9	2.16	2.1	0.50	1.8	0.49
												
2.5	1.50	0.6	0.50	0.4	0.41	13-14	7.4	2.02	2.0	0.52	1.7	0.58
1.4	1.15	0.2	0.30	0.1	0.24	14-15	6.0	2.56	1.5	0.79	1.1	0.68
0.7	0.79	0.1	0.20	0.0	0.14	15-16	3.5	2.37	0.8	0.73	0.5	0.77
(0.4)	(0.58)	(0.0)	(0.06)	(0.0)	(0.04)	16-17	1.8	1.74	0.3	0.38	0.2	0.25
(0.2)	(0.46)	(0.0)	(0.00)	(0.0)	(0.00)	17-18	0.9	1.04	0.1	0.17	0.0	0.08

Figures in parenthesis based on 35-44 children only, as data were not available on every subject at these ages,

* Maximum variation shown by bold figures.

Finally, during the process of a lower limb transtibial amputation, the ideal distance at skeletal maturity from the medial knee joint should be 15 cm (Table 5). In order to avoid sharp corners, the tibial crest should be chamfered. A myodesis of calf muscles to the periosteum is performed along with trimming the nerves proximally. Ideally the skin flaps should be skewed to avoid weight bearing points and to achieve a favorable shape for early prosthetic fitting. (Table 4)

As discussed previously, it is essential to have an adequate length in the stump in order to apply the prosthesis. Stumps can be lengthened via the Ilizarov technique. Up to sixty percent lengthening was achieved by Orhun and colleagues without major complications in cases with forearm amputations.^18^ In another study, Alekberov et al. described a mean lengthening of 5.6cm in the case of below-elbow amputation stumps in six patients.^19^

*Tissue Coverage*

If primary skin apposition cannot be achieved at closure then other reconstructive techniques need to be considered. These include local flaps, free flaps and even tissue expansion to create expandable flaps. These techniques have been successful with function and form, allowing both weight bearing and improvement in the appearance of the stump.^20^^,^^21^ A full discussion of reconstructive techniques is beyond the scope of our paper.

The time from traumatic amputation to fitting of the definitive prosthesis is very important in order to ensure an optimal outcome for the stump. In the case of transtibial amputations, the different types of dressing include simple soft gauze dressings, thigh rigid cast dressings, shorter removable rigid dressings and prefabricated pneumatic dressings.^22^ Postoperative prosthetic attachments can be added to all but simple soft dressings. The advantages of removable rigid dressing are that they protect the stump from trauma are safe, cost effective, reduce skin breakdown and reduce distal edema.^23^^-^^25^ They have no proven benefit over soft dressing in terms of healing, analgesic requirements or hospital stay.^26^^,^^27^

*Rehabilitation*

The psychological impact that amputation has on the pediatric amputee is one of the most important factors and must be addressed if rehabilitation is to be successful. Desmond et al reported a presence of significant depressive symptoms in 28.3% of patients and significant anxiety symptoms in 35.5% of these patients, as measured by the Hospital Anxiety and Depression Scale, Depression subscale (HADS-D).^9^ Post-traumatic Stress Disorder is the most common psychiatric disorder experienced after such traumatic events and can be observed in not only the pediatric amputee but also in their primary care taker, which ultimately has an impact upon the overall rehabilitation of the child.^28^ Cognitive Behavioral Therapy (CBT) has been shown to be the best treatment approach towards children who experience traumatic-related symptoms which may be augmented with family support.^29^ It is important to note that often it is the traumatic incidence which causes the amputation, rather than the amputation itself, which has the greatest impact upon the patient. Boyle et al reported that those who had amputations as a result of malignancy better adapted to disability when compared to amputations due to trauma.^30^ Coping styles are important predictors of psychosocial adaptation, with avoidance being strongly associated with emotional anguish and poor adjustment.^31^^,^^32^

It is important to consider that pediatric population copes well with both physical and psychological stressors and thus have a faster recovery.^32^ Also pediatric population has been seen to adapt well to changes using various coping mechanisms.^32^ The approach to these patients, in terms of post-amputation rehabilitation should be individualized. 

*Late Complications*

Late complications include soft tissue lesions, exostosis, painful neuroma and phantom limb pain.^33^ Any necrotic debris should be debrided aggressively and immediate wedge resection or re-amputation maybe warranted in such cases. Neuromas can result in pain on traction from scar tissue and can be prevented by dividing the nerves at a more proximal level, allowing them to retract away from the end of the stump and avoid entrapment in the scar tissue. Neuromas can be further divided surgically at a proximal level to relieve severe pain. Phantom pain usually disappears with regular use of the prosthesis, pain management and adequate counseling. 

A significant problem with traumatic pediatric amputations is also the osseous overgrowth which can lead to skin perforation, pressure ulcers and difficulty with prosthesis fitting. Consequently, numerous surgical revisions may be required secondary to the consequences of osseous overgrowth.^34^ This is in stark comparison to joint disarticulation which never develops overgrowth. Osseous overgrowth can be reduced by capping the stump with autologous material from the injured limb as compared to resection and revision or the use of a silastic bung.^35^

## CONCLUSION

Traumatic limb amputations in the pediatric age group can result in disability and can have psychological consequences on the amputee. A multidisciplinary treatment approach is advocated with input from a physiotherapist, nursing staff, child psychiatrist and the surgeon. Parental support and counseling are necessary to help identify coping mechanisms that are effective for the amputee and the family. The key is to preserve as much residual limb length as possible in order to ensure a favorable outcome with the application of prosthesis after full growth of the limb has taken place. Complications albeit few, can be disabling and need regular follow up and surgical revisions, if the need arises.
